# Outcomes associated with participation in a sustainability environmental education program: preschool children’s life skills and computational thinking

**DOI:** 10.3389/fpsyg.2026.1820824

**Published:** 2026-05-20

**Authors:** Merve Arabacı, Mehmet Oğuz Göle, Ahmet Murat Uzun, Ümit Ünsal Kaya, Nezahat Hamiden Karaca

**Affiliations:** 1 Department of Early Childhood Education, Afyon Kocatepe University, Afyonkarahisar, Türkiye; 2 Department of Instructional Technologies, Afyon Kocatepe University, Afyonkarahisar, Türkiye; 3 School of Foreign Languages, Afyon Kocatepe University, Afyonkarahisar, Türkiye

**Keywords:** computational thinking, early childhood education, life skills, nature-based learning, sustainability

## Abstract

**Introduction:**

This study examined whether participation in the Sustainable Environmental Education Program (SEEP) was associated with differences in preschool children’s life skills and computational thinking-related outcomes.

**Methods:**

A quasi-experimental pretest-posttest design with a non-equivalent control group was employed. Intact classrooms were allocated to either an experimental condition (*n* = 112) or a control condition (*n* = 110) receiving standard preschool programming. Participants were 222 children aged 61–72 months from 12 public preschool classrooms in a mid-sized city in Türkiye. A three-week follow-up assessment was also conducted in the experimental group. Teachers completed the Early Childhood Life Skills Scale, and children were individually assessed using the TechCheck-K mathematical thinking and critical thinking dimensions. SEEP consisted of 20 sessions delivered over 10 weeks and was organized around four thematic units using recyclable and natural materials.

**Results:**

Relative to the control group, children in the experimental group showed more favorable pretest-to-posttest patterns in overall life skills scores and in TechCheck-K mathematical thinking and critical thinking scores. Within the experimental group, scores did not change significantly over the three-week follow-up period.

**Discussion:**

These findings provide preliminary evidence that participation in a sustainability-oriented experiential preschool program may be associated with selected socio-emotional and cognitive outcomes in early childhood. Interpretation is constrained by the quasi-experimental design, classroom-level assignment analyzed at the individual level, reliance on teacher-reported life skills ratings, and a short follow-up conducted only within the experimental group.

## 1 Introduction

Sustainability refers to meeting present needs while protecting natural resources for future generations and sustaining life within planetary limits (UNESCO, 2010). Over time, the concept of sustainability has expanded from a primarily environmental concern to a broader framework reflected in educational goals and practices. Within this perspective, education is seen as an important means of fostering the knowledge, skills, attitudes, and values needed to support a more sustainable society (Rieckmann, 2017). Environmental sustainability, in particular, involves using natural resources within regenerative limits and reducing waste in ways that do not damage ecosystems (Holden et al., 2014).

Sustainability education extends the scope of environmental education by not only promoting scientifically grounded understandings of ecosystems but also encouraging ethical relations with nature and social–political appraisal of environmental issues. Environmental education remains a key foundation for achieving these aims (Davis and Elliott, 2014). Early environmental education aims to heighten sensitivity toward nature, enable recognition of environmental problems, and build problem-solving capacities (Borg et al., 2017; Davis, 2010).

Early childhood is a critical period for cognitive, social, and emotional development and is also formative for environmental attitudes, values, and behaviors. Accordingly, scholars emphasize the importance of introducing sustainability-oriented environmental education in the early years (Ardoin and Bowers, 2020; Ärlemalm-Hagsér and Sandberg, 2017). In practice, such education should provide direct contact with nature and opportunities to engage with natural materials. These experiences can support multiple domains of development by helping children build a sense of connection with nature, develop sensitivity to environmental issues, and strengthen pro-environmental attitudes and behaviors (Chawla, 2020; Kuo et al., 2019). They may also contribute to cognitive development by encouraging curiosity, exploration, and problem solving. In this respect, sustainability-oriented experiences may also help lay groundwork for computational thinking in early childhood (Ernst and Burcak, 2019).

Computational thinking (CT) involves the use of systematic and algorithmic reasoning to analyze problems and develop solutions (Wing, 2006; Grover and Pea, 2013). In early childhood, CT is associated with skills such as logical reasoning, sequencing, pattern recognition, and problem solving, which are relevant to both academic learning and everyday situations. Environmental challenges, by their nature, often require interdisciplinary and structured ways of thinking, making CT a potentially valuable component of sustainability education (Christensen, 2023). Emerging studies have begun to illustrate this intersection. For example, Kanaki et al. (2025) reported that Bee-Bot activities supported both young children’s algorithmic thinking and their environmental awareness. In addition, nature-based experiences may also contribute to processes related to CT by supporting problem solving, creative thinking, and executive functions (Ernst and Burcak, 2019; Om et al., 2023).

CT is not limited to technical skills; in early childhood, it is closely related to reasoning, problem solving, and flexible thinking in meaningful contexts. In sustainability-oriented learning environments, these processes may also support broader life skills such as communication, collaboration, self-regulation, and decision making. Life skills begin to develop in the early years and play an important role in socio-emotional development (Banning and Sullivan, 2010). In this respect, interactions with natural settings may provide rich opportunities for supporting children’s life-skill development (Ernst and Theimer, 2011).

When linked with sustainability education, life skills may include sensitivity to nature, responsibility-taking, and environmentally responsible behavior (Davis and Elliott, 2014). Children who learn about recycling, energy saving, and using resources efficiently may incorporate such knowledge into their daily routines and begin to develop pro-environmental habits. Studies suggest that young children can engage with environmental problems in ways that involve both awareness and responsibility. For example, garden-based activities have been associated with communication, emotion management, initiative taking, and discussions about local biodiversity protection (Sageidet et al., 2019). Other research has shown that children can develop environmental responsibility through practices such as litter collection, recycling, and conserving water and energy (Melis et al., 2020). In this sense, sustainability education may support competencies such as critical thinking, collaboration, and active citizenship while also strengthening environmental awareness and responsibility (Hedefalk et al., 2015). These intersections suggest that sustainability-oriented experiences may also provide a meaningful context for connecting life skills with computational thinking (Elliott and Davis, 2018).

Despite growing interest, few studies have explicitly examined the relationship between sustainability-oriented environmental education and CT in early childhood, and the broader evidence base remains thin. Much of the existing CT research in early childhood has focused on specialized tools and digital platforms rather than sustainability-oriented learning contexts (McCormick and Hall, 2022). Some recent studies suggest possible points of integration. For example, Kanaki et al. (2025) used Bee-Bot activities to support algorithmic thinking and environmental awareness, while Yang et al. (2024) and Leung et al. (2024) explored digital approaches related to sustainability and CT. By contrast, studies linking sustainability education to life skills have more often focused on gardens, nature-based activities, and everyday environmental practices (Sageidet et al., 2019; Melis et al., 2020; Qu et al., 2025). Overall, this pattern points to a gap in research at the intersection of sustainability education, life skills, and CT in early childhood (Kanaki and Kalogiannakis, 2023).

This study responds to these gaps by examining whether participation in a Sustainable Environmental Education Program (SEEP) is associated with differences in life skills and computational thinking-related outcomes in early childhood. Unlike many previous studies that have focused on programmable tools or digital platforms, SEEP emphasizes activities using recyclable and natural materials and engages children with sustainability-related problems in everyday contexts. In this respect, the study aims to contribute to the limited literature examining life skills and computational thinking together within a sustainability-oriented preschool program and to explore patterns of short-term change following the posttest assessment. Building on this body of work, the present study examines whether participation in the Sustainable Environmental Education Program (SEEP) is associated with differences in preschool children’s life skills and TechCheck-K mathematical and critical thinking scores. It also examines what pattern of short-term change, if any, is observed in the experimental group over the three-week follow-up period. To guide this inquiry, the following research questions were posed:

*RQ1*. Is participation in a sustainable environmental education program associated with differences in early childhood students’ life skills?

*RQ2*. Is participation in a sustainable environmental education program associated with differences in early childhood students’ computational thinking-related skills, measured through mathematical and critical thinking scores?

*RQ3*. What pattern of short-term change, if any, was observed in life skills and computational thinking scores within the experimental group over the three-week follow-up period?

## 2 Materials and methods

### 2.1 Research design

This study employed a quasi-experimental, classroom-allocated pretest-posttest design with a non-equivalent control group, supplemented by a three-week follow-up assessment in the experimental group (Fraenkel and Wallen, 2019). Intact preschool classrooms, rather than individual children, were assigned to the study conditions in order to preserve the natural classroom structure. Life skills and computational thinking were assessed at pretest and posttest in both groups. In addition, a follow-up assessment was conducted 3 weeks after the intervention in the experimental group in order to examine the short-term maintenance of posttest scores. Random assignment of individual children to experimental and control conditions was not pursued in this study, as it would have required splitting naturally formed classroom groups, which raises well-documented ethical and practical concerns in early childhood educational research (Shadish et al., 2002; Cook and Campbell, 1979). Classroom-level allocation to intact groups is a common and feasible approach in school-based quasi-experimental research and was selected here in order to preserve the natural instructional context while still allowing comparison with a non-equivalent control group (Gersten et al., 2005). Given the quasi-experimental design, classroom-level allocation analyzed without multilevel modeling, and the single-city sample, the present study should be interpreted as an exploratory investigation. The findings therefore provide initial, hypothesis-generating evidence rather than confirmatory evidence of program effectiveness.

### 2.2 Participants

The study initially involved 230 children enrolled in 12 public preschool classrooms in a mid-sized city in Türkiye. Six classrooms were allocated to the experimental condition and six classrooms to the control condition. The classrooms were selected from neighborhoods representing broadly comparable socioeconomic conditions, with each condition including classrooms from lower-income and middle-income areas.

The final analytical sample consisted of 222 children, including 110 in the control group and 112 in the experimental group, after data screening procedures for missing data and outlier values. All children remained in the study during the intervention period; however, a small number of cases were excluded from the final analyses because their data did not meet the predefined screening criteria.

Children were between 61 and 72 months of age and showed typical developmental characteristics. To reduce interaction between the study conditions within the same institutions, the experimental classrooms were scheduled in the morning and the control classrooms in the afternoon where applicable. Although this arrangement was intended to minimize contamination, it may also have introduced a time-of-day difference between the conditions. Purposeful criterion sampling was used. Children were required to be enrolled in a public independent preschool, to fall within the target age range, and to participate regularly enough to complete the study procedures. Information regarding previous exposure to coding- or computational thinking-related activities was collected descriptively through the personal information form and is reported in Table 1. No child withdrew during the intervention period. However, not all cases were retained in the final statistical analyses because exclusions were applied at the data-screening stage for incomplete data and outlier values. In addition, follow-up analyses in the experimental group were based on the children who were available on the day of the follow-up assessment.

**Table 1:** Demographic characteristics of the participants.

Variable	Category	Control		Experimental	
		*n*	%	*n*	%
Gender	Female	48	43.6	44	39.3
	Male	62	56.4	68	60.7
Years in preschool	1 year	34	30.9	62	55.4
	2 years	56	50.9	38	33.9
	3 years	20	18.2	12	10.7
Number of siblings	1	66	60.0	43	38.4
	2	16	14.5	39	34.8
	3	12	10.9	14	12.5
	4 or more	16	14.5	16	14.3
Mother’s age (years)	20–25	1	0.9	11	9.8
	26–30	29	26.4	44	39.3
	31–35	38	34.5	33	29.5
	41 and above	42	38.2	24	21.4
Father’s age (years)	20–25	0	0.0	4	3.6
	26–30	7	6.4	29	25.9
	31–35	36	32.7	38	33.9
	41 and above	67	60.9	41	36.6
Mother’s educational level	Primary school	13	11.8	13	11.6
	Middle school	13	11.8	27	24.1
	High school	24	21.8	37	33.0
	Associate degree	25	22.7	9	8.0
	Bachelor’s degree	31	28.2	19	17.0
	Postgraduate degree	4	3.6	7	6.2
Father’s educational level	Primary school	5	4.5	7	6.2
	Middle school	17	15.5	24	21.4
	High school	18	16.4	44	39.3
	Associate degree	22	20.0	9	8.0
	Bachelor’s degree	35	31.8	20	17.9
	Postgraduate degree	13	11.8	8	7.1
Previous coding education	Yes	7	6.4	15	13.4
	No	103	93.6	97	86.6

Table 1 presents the demographic characteristics of the participants. The sample was relatively balanced by gender, with slightly more boys than girls in both groups. Most children had one sibling, and the majority of parents were in the 31–41 age range. Parents’ education levels varied, ranging from primary school to postgraduate degrees, which provided socioeconomic diversity in the sample. No significant pre-intervention differences were observed between the experimental and control groups across demographic variables (all *p* > 0.05).

### 2.3 Instruments

#### 2.3.1 Personal information form

A researcher-developed personal information form was used to collect descriptive information on children’s gender, number of siblings, years in preschool, parents’ age and educational level, and previous exposure to coding- or computational thinking-related activities.

#### 2.3.2 Computational thinking skills assessment tool (TechCheck-K)

CT skills were assessed using TechCheck-K, developed by Relkin and Bers (2021) and adapted into Turkish by Yılmaz and Yılmaz Uysal (2024). This multiple-choice assessment is designed for children aged 5–6 years and measures developmentally appropriate CT concepts in an unplugged format. The Turkish version consists of 14 items grouped under two dimensions: mathematical thinking (11 items) and critical thinking (3 items). In the present study, analyses focused on these two dimension scores rather than a single overall score. The tasks include sequencing, shortest-path puzzles, missing-symbol series, object ordering, obstacle mazes, symbol–shape matching, recognition of technological concepts, and symmetry problems. Each item presents three visual response options and is scored dichotomously (1 = correct, 0 = incorrect). The instrument was administered individually by trained researchers in a quiet room within the preschool setting using a standardized administration procedure, and administration took approximately 7 min per child. In the Turkish adaptation study, internal consistency was reported as KR-21 = 0.72, indicating acceptable reliability for research purposes. Because the instrument yields dimension-specific scores, mathematical thinking and critical thinking were analyzed separately in the inferential analyses. It should be noted that the critical thinking dimension comprises only three items (maximum possible score = 3). This restricted score range limits variability and may render the critical thinking subscale susceptible to ceiling effects, particularly in higher-performing groups. Accordingly, findings related to this dimension should be interpreted with additional caution.

#### 2.3.3 Early childhood life skills scale

Life skills were measured using the Early Childhood Life Skills Scale (ECLSS) developed by Topçu Bilir (2022). The scale consists of 56 Likert-type items scored from 1 (never) to 5 (always), with higher scores indicating stronger life skills. It includes items related to communication, interpersonal relationships, critical and creative thinking, problem solving, decision making, emotion regulation, coping with stress, self-awareness, empathy, and health and safety skills. Confirmatory factor analysis results reported in the scale-development study supported its validity, and the instrument was shown to be reliable for use with Turkish children (Topçu Bilir, 2022). In the present study, classroom teachers completed the scale for each child on the basis of their routine classroom observations, and the total ECLSS score was used in the statistical analyses. Internal consistency in the present sample was high (Cronbach’s α = 0.88). In the present study, the scale was completed by classroom teachers who were able to observe the children on a daily basis. Although teacher ratings allowed the assessment of children across everyday classroom contexts, reliance on teacher-reported data may have introduced subjective bias. In addition, teachers were aware of classroom participation in the intervention, so expectancy effects cannot be ruled out.

#### 2.3.4 Fidelity of implementation

The intervention was implemented by a team of four researchers with formal training in early childhood education at the undergraduate and/or graduate level and extensive classroom teaching experience (*M* = 8.2 years, range = 5–12 years). Prior to implementation, the research team participated in a two-day training workshop designed to promote consistency across implementers. The workshop included: (a) a review of the theoretical foundations of the program, including constructivism, experiential learning, sustainability education, and computational thinking; (b) hands-on practice with all 20 activities; (c) role-play of instructional scenarios to develop questioning techniques and scaffolding strategies; and (d) calibration exercises to support consistency in implementation across classrooms. To enhance procedural consistency, each session was guided by a detailed written lesson plan (3–4 pages per session). These plans specified the session objectives, required materials and preparation procedures, step-by-step instructional sequence with approximate timing, key questions to promote discussion and reasoning, anticipated child responses and corresponding instructional moves, differentiation strategies for varying developmental levels, and documentation procedures.

Implementation fidelity was monitored using structured observation checklists developed for this study. Fidelity observations were completed by a member of the research team who was not leading the observed session. Each checklist consisted of 15 items assessing three domains: (a) use of the specified materials (5 items), (b) completion of the planned activity components (5 items), and (c) implementation of key instructional strategies, such as open-ended questioning and small-group facilitation (5 items). Each item was rated as “implemented,” “partially implemented,” or “not implemented.” Across the 20 sessions delivered in 6 experimental classrooms (120 total session instances), fidelity observations were completed for 42 sessions (35% of all sessions). Each classroom was observed at least once within each thematic unit, yielding a minimum of four fidelity observations per classroom. Fidelity documentation indicated that the planned materials and core activity components were fully implemented in 94.2% of the observed sessions, calculated as the proportion of checklist items rated as fully implemented across observed sessions. Rates ranged from 89 to 98% across classrooms. When deviations occurred, they typically involved minor adjustments to timing, such as extending the exploration phase when children were highly engaged, rather than omission of core intervention components.

In addition, the researchers maintained reflective journals throughout the implementation period to document practical issues, child engagement, and any adaptations made during sessions. The implementation team also met weekly (60–90 min) to review challenges, discuss instructional decisions, and ensure that any adaptations remained aligned with the core principles of the program. Although formal inter-rater reliability was not assessed, the combination of shared training, detailed lesson planning, structured fidelity documentation, and weekly calibration meetings was intended to support implementation consistency across classrooms.

### 2.4 Sustainable environmental education program

The Sustainable Environmental Education Program (SEEP) was developed by the researchers as a structured preschool intervention integrating sustainability-oriented environmental education, hands-on exploration, and developmentally appropriate problem-solving experiences. The program was grounded in constructivist and experiential learning theories. Its theoretical framework drew on Vygotsky’s emphasis on scaffolding and social interaction, Piaget’s emphasis on active learning through interaction with the environment, and Dewey’s view of direct experience as central to meaningful learning (Dewey, 1938; Piaget, 1972; Vygotsky, 1978). In addition, the program was informed by UNESCO’s (2017) view that early childhood environmental education should foster not only knowledge acquisition but also values, attitudes, participation, and everyday responsibility.

The overall study process lasted 14 weeks. Pretest measures were administered during the first 2 weeks. The intervention itself was implemented over 10 weeks and consisted of 20 sessions delivered twice per week. Each session lasted approximately 40–50 min. Posttest measures were collected during the following 2 weeks. A follow-up assessment was administered 3 weeks after the completion of the intervention in the experimental group in order to examine the short-term maintenance of posttest scores.

SEEP was organized around four thematic units. The first unit, Nature and Cycles, focused on helping children observe natural processes, change over time, and relationships within the environment through activities such as composting, handmade paper production, birdhouse-related design tasks, rainwater collection, and soilless growing practices. The second unit, Energy and Resources, focused on the use, conservation, and renewal of resources through activities related to solar and wind energy, natural filtration, and water conservation. The third unit, Environmental Problems and Solutions, engaged children in identifying environmental problems such as air, water, soil, noise, and light pollution and in discussing possible responses through collaborative inquiry. The fourth unit, Sustainable Life Practices, emphasized daily routines and responsibilities related to recycling, saving energy and water, sharing materials, and taking responsibility for tasks at home and at school.

Each session followed a consistent three-phase structure designed to scaffold children’s participation and understanding. In Phase 1 (Opening Circle; approximately 10 min), the researcher introduced the theme of the day through whole-group discussion, activated prior knowledge, and used visual or concrete prompts such as photographs, natural objects, or children’s previous work. In Phase 2 (Exploration and Activity; approximately 25–30 min), children worked individually or in small groups, depending on the task, to manipulate materials, test ideas, observe environmental phenomena, and represent their thinking through drawings, dictation, or emergent writing. During this phase, the researcher circulated among children, posed guiding questions, provided differentiated support, and encouraged peer interaction and discussion. In Phase 3 (Reflection and Closure; approximately 10 min), the whole group reconvened to share observations, compare findings, identify patterns, and discuss possible links between the activity and everyday life.

Computational thinking-related processes were intentionally supported through the instructional design of the activities. For example, complex environmental problems were broken down into smaller, more manageable parts to support problem decomposition; children were encouraged to identify regularities and changes over time to support pattern recognition; and multi-step tasks were used to support sequencing and procedural reasoning. In activities such as water filtration, children were encouraged to plan and test the order of materials, observe the outcome, and revise their procedure when needed. Throughout the program, researchers used open-ended questions to make these forms of thinking more explicit, such as asking children what steps they had followed, what patterns they noticed, and what might happen if part of the process were changed.

The translation of these principles into practice can be illustrated with several examples. In the composting activity within the Nature and Cycles unit, children first observed prepared compost materials, then created simple mini-composters using transparent containers, and subsequently documented visible changes over time through repeated observation. In the Energy and Resources unit, children explored solar energy through simple child-safe models using small photovoltaic components, wires, and LED lights, and compared how light conditions affected the outcome. In the Environmental Problems and Solutions unit, children examined visual materials related to air pollution, discussed possible effects on living things, and generated both individual and community-level responses.

All activities were designed to be feasible for preschool settings and to model sustainability principles through the use of accessible, low-cost materials. Natural materials included soil, water, seeds, leaves, twigs, rocks, sand, and vegetable scraps. Recyclable materials included plastic bottles, cardboard boxes, egg cartons, newspaper, and fabric scraps. Purchased materials were limited to a small number of low-cost items, such as small photovoltaic cells, LED lights, thermometers, and magnifying glasses.

#### 2.4.1 Control condition

The control condition comprised six intact classrooms (*n* = 110 children) that continued with the standard preschool curriculum throughout the study period. Control-group children were assessed at the same time points as experimental-group children (pretest and posttest) using the same instruments and procedures. No SEEP activities, supplementary sustainability content, or structured computational thinking program was introduced in the control condition. Routine classroom practices may have included general thematic activities related to daily life, nature, or the environment; however, these activities were not designed to mirror the structure, duration, or pedagogical approach of the experimental program. The absence of an attention-matched active control condition is therefore acknowledged as a limitation when interpreting group differences.

### 2.5 Data analysis

Prior to the main analyses, the dataset was screened for missing data, univariate outliers, and multivariate outliers. Standardized scores, boxplots, and Mahalanobis distance values were examined for this purpose. Cases identified during data screening were excluded from the final analytical sample according to the predefined cleaning criteria. Descriptive statistics and Pearson correlation coefficients were first examined to provide an overview of the distributions and associations among the study variables. To address Research Question 1, changes in life skills scores from pretest to posttest were examined using a mixed repeated-measures approach, with group (experimental vs. control) as the between-subjects factor and time (pretest vs. posttest) as the within-subjects factor. Because some assumptions relevant to repeated-measures analysis were not fully met, the multivariate approach to repeated measures was used and Pillai’s Trace was prioritized where appropriate (Pallant, 2010; Tabachnick and Fidell, 2013). To address Research Question 2, a mixed-design repeated-measures MANOVA was conducted with group (experimental vs. control) as the between-subjects factor and time (pretest vs. posttest) as the within-subjects factor. The two dependent variables were the mathematical thinking and critical thinking dimension scores derived from TechCheck-K.

To address Research Question 3, follow-up analyses were conducted only within the experimental group because follow-up data were not collected from the control group. Accordingly, these analyses were intended to examine short-term within-group change following posttest in the intervention group rather than comparative long-term intervention effects or retention. For life skills, posttest and follow-up scores in the experimental group were compared using a paired-samples *t*-test. For computational thinking, short-term maintenance was examined within the experimental group using a repeated-measures multivariate analysis across the mathematical thinking and critical thinking dimensions. Because children were nested within classrooms, the findings were interpreted with caution in relation to possible intra-class dependency. However, the limited number of classrooms did not permit multilevel modeling, and the primary analyses were therefore conducted at the individual level. Statistical assumptions relevant to the planned analyses were examined prior to inferential testing. Homogeneity of variance was assessed using Levene’s test, and homogeneity of covariance matrices was evaluated using Box’s M test where appropriate. When assumption violations were identified, more robust multivariate test statistics such as Pillai’s Trace were prioritized (Pallant, 2010; Tabachnick and Fidell, 2013). Bonferroni adjustments were applied for multiple comparisons where relevant. Statistical significance was evaluated at the 0.05 level unless a more conservative threshold was required. Effect sizes were reported using partial eta squared (ηp^2^) for the repeated-measures and multivariate models and Cohen’s d for paired-samples *t*-tests where applicable. Because children were nested within classrooms but the limited number of classrooms did not permit multilevel modeling, the inferential analyses were conducted at the individual level and interpreted with caution in relation to possible classroom-level dependency (Table 2).

**Table 2:** Descriptive statistics and Pearson correlations.

Variable	*M*	SD	1	2	3	4	5
1. Life skills (Pre)	236.50	36.73					
2. Life skills (Post)	254.74	32.16	0.490**				
3. Math test (Pre)	4.51	1.72	0.015	−0.009			
4. Critical test (Pre)	2.16	0.89	0.272**	0.039	0.192**		
5. Math test (Post)	6.98	1.87	0.083	0.123	0.376**	0.213**	
6. Critical test (Post)	2.75	0.52	0.139*	0.154*	0.133*	0.237**	0.292**

*p < 0.05, **p < 0.01.

## 3 Results

This section presents the findings in relation to the research questions. First, descriptive statistics and Pearson correlations are reported to provide an overview of the study variables. Next, the pretest-to-posttest findings for life skills and TechCheck-K outcomes are presented. Finally, follow-up findings from the experimental group are reported to examine short-term posttest-to-follow-up change over the three-week period. According to the results of the Pearson correlation analysis conducted to examine the relationships among the variables, a significant positive relationship was found between life skills and critical thinking (*r* = 0.22, *p* < 0.01). In contrast, no significant relationship was found between life skills and mathematical thinking (*r* = 0.05, *p* > 0.05). In addition, mathematical thinking was also found to be positively and significantly related to critical thinking (*r* = 0.24, *p* < 0.01). For each group, the mean, standard deviation, skewness, and kurtosis values are presented in Table 3.

**Table 3:** Descriptive statistics according to groups.

Measure	Group	*M*	SD	Skewness	Kurtosis
Life skills (Pre)	Control	236.50	36.73	−0.999	0.500
	Experimental	237.85	34.23	−0.759	−0.228
Life skills (Post)	Control	234.22	31.48	−1.138	1.250
	Experimental	254.74	32.16	−1.687	1.986
Math test (Pre)	Control	4.51	1.72	0.183	−0.020
	Experimental	4.86	1.65	0.122	−0.183
Critical test (Pre)	Control	2.16	0.89	−0.798	−0.191
	Experimental	1.98	0.87	−0.566	−0.291
Math test (Post)	Control	4.78	1.82	0.356	0.065
	Experimental	6.98	1.87	−0.094	−0.450
Critical test (Post)	Control	2.38	0.80	−0.807	−0.946
	Experimental	2.75	0.52	−1.929	2.948

The *first research question* examined whether participation in the sustainable environmental education program was associated with differences in children’s life skills.

Using the multivariate approach to repeated measures, changes in life skills scores from pretest to posttest were examined with group (experimental vs. control) as the between-subjects factor and time (pretest vs. posttest) as the within-subjects factor. Prior to the analysis, homogeneity of variance was assessed using Levene’s test, and the assumption was found to be met for both the pretest (*F*(1, 220) = 0.371, *p* = 0.543) and the posttest (*F*(1, 220) = 0.250, *p* = 0.618). However, the results of Box’s M test indicated a violation of the assumption of homogeneity of covariance matrices (Box’s *M* = 35.498; *F* = 11.716; *p* < 0.001). Therefore, Pillai’s Trace, which is considered a more robust test statistic, was used in the analyses. The results showed a significant main effect of time on life skills scores (*F*(1, 220) = 10.54, *p* = 0.001, partial η^2^ = 0.046). A comparison of the pretest mean (*M* = 237.17, SE = 2.38) and posttest mean (*M* = 244.48, SE = 2.14) indicated an increase in scores over time.

The main effect of group was also significant (*F*(1, 220) = 7.75, *p* = 0.006, partial η^2^ = 0.034). The mean score of the experimental group (*M* = 246.29, SE = 2.79) was higher than that of the control group (*M* = 235.36, SE = 2.76). In addition, the interaction effect between time and group was statistically significant (*F*(1, 220) = 18.14, *p* < 0.001, partial η^2^ = 0.076).

**Figure 1:**
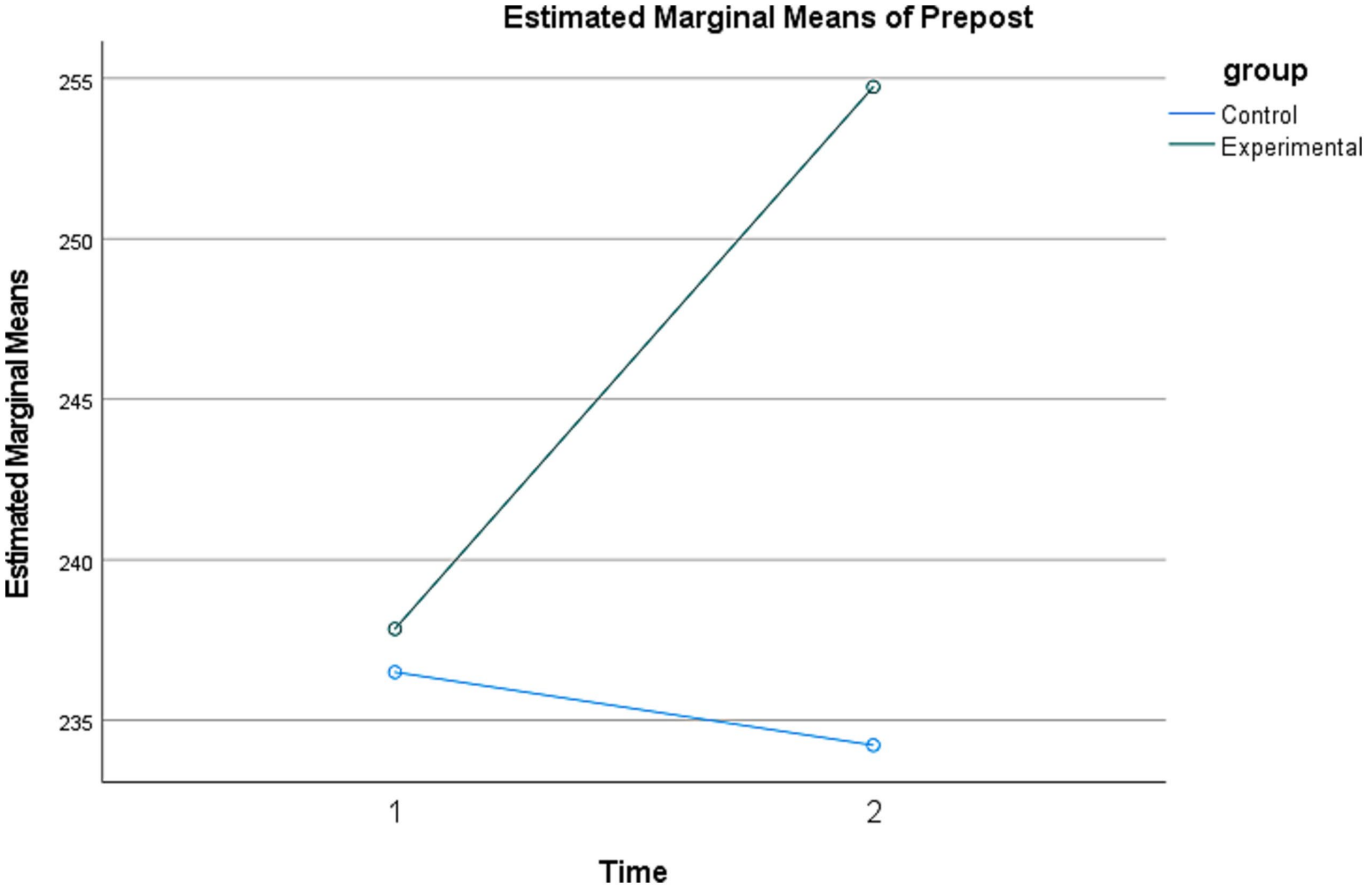
Mean change in life skills over time across groups.

To examine changes within each group, paired-samples *t*-tests with Bonferroni correction were conducted (0.05/2 = 0.025). The results showed no statistically significant pretest-to-posttest change in life skills scores in the control group (mean difference = −2.28). In contrast, the experimental group showed a statistically significant increase in life skills scores (mean difference = 16.89, *p* < 0.001). These within-group findings should be interpreted alongside the significant time × group interaction reported above. Changes between the groups are presented visually in Figure 1.

The *second research question* examined whether participation in the sustainable environmental education program was associated with differences in children’s computational thinking-related skills. As part of the analyses related to Table 3, a mixed-design repeated-measures MANOVA was conducted to examine whether computational thinking skills differed as a function of time and group. In the analysis, mathematical thinking and critical thinking skills were treated as the dependent variables, whereas time (pretest and posttest) and group (experimental and control) were specified as the independent variables. Prior to the analysis, the assumptions of homogeneity of variance and covariance were evaluated using Box’s M and Levene’s tests. The results showed that Box’s M test was significant (*p* < 0.05) and that Levene’s test yielded partially significant results. This indicated that the variance–covariance matrices were not equal across groups. However, given the adequate sample size and the robustness of MANOVA to violations of assumptions, the analysis was continued. Accordingly, Pillai’s Trace, which is considered a more robust test statistic, was used. In addition, a more conservative significance level (0.01) was adopted (Pallant, 2010; Tabachnick and Fidell, 2013).

**Figure 2:**
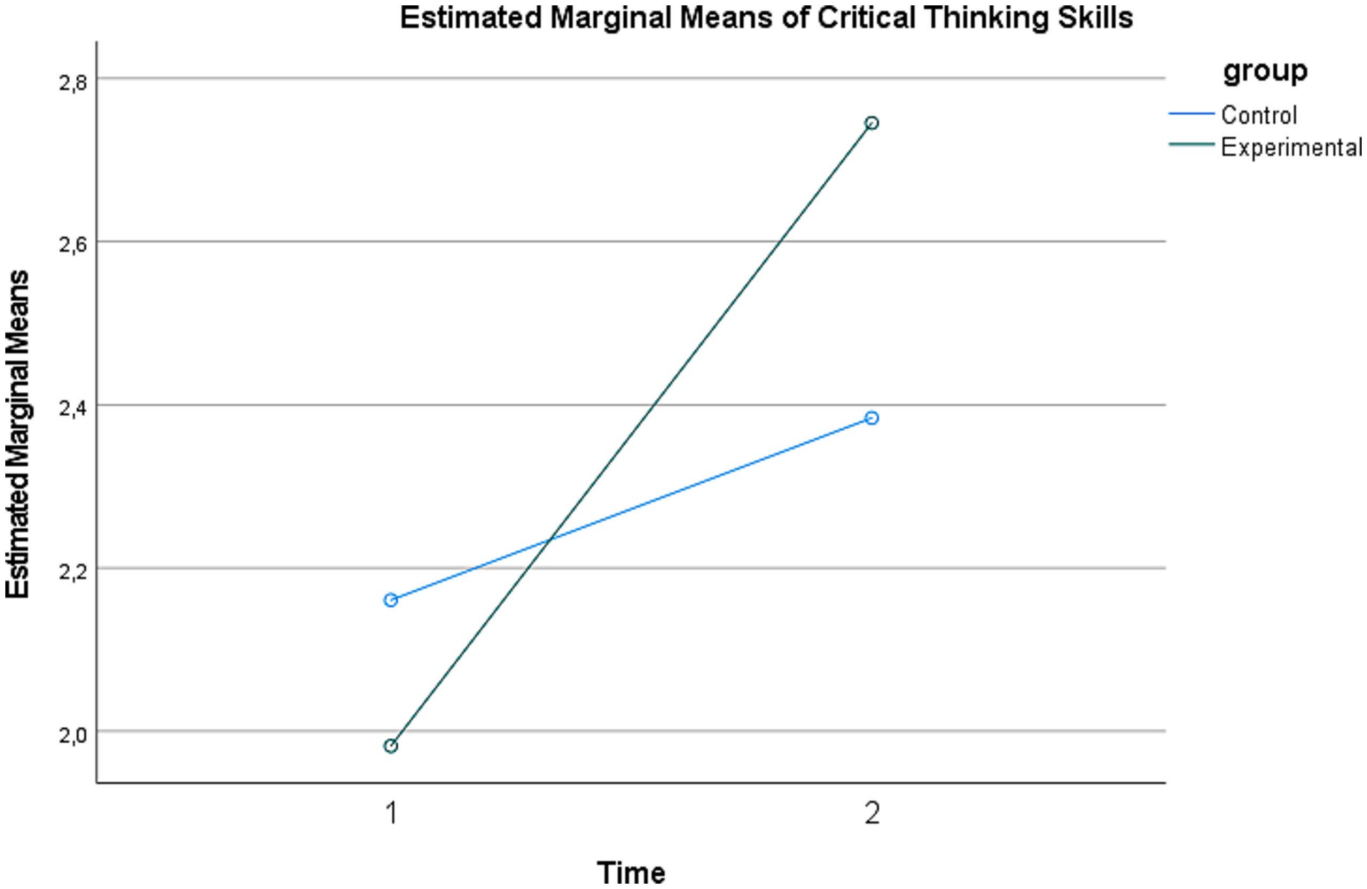
Mean change in critical thinking across groups.

The results revealed a significant main effect of time (Pillai’s Trace = 0.383, *F*(1, 220) = 136.78, *p* < 0.001, partial η^2^ = 0.383). A comparison of the pretest mean (*M* = 3.38, SE = 0.07) and the posttest mean (*M* = 4.22, SE = 0.07) indicated an increase in scores over time. The main effect of group was also significant (*F*(1, 220) = 33.45, *p* < 0.001, partial η^2^ = 0.132). The mean score of the experimental group (*M* = 4.14, SE = 0.08) was higher than that of the control group (*M* = 3.46, SE = 0.08). The interaction effect between time and group was also found to be significant (Pillai’s Trace = 0.238, *F*(1, 220) = 68.72, *p* < 0.001, partial η^2^ = 0.238) (Figure 2).

The analyses further indicated that the pattern of change over time differed across the two TechCheck-K dimensions as a function of group (Pillai’s Trace = 0.080, *F*(1, 220) = 19.15, *p* < 0.001, partial η^2^ = 0.080). This finding suggests that the magnitude of pretest-to-posttest change differed across the mathematical thinking and critical thinking dimensions between the experimental and control groups. According to the Bonferroni-adjusted multiple comparisons, both mathematical thinking (mean difference = 2.12, *p* < 0.001) and critical thinking (mean difference = 0.76, *p* < 0.001) increased significantly in the experimental group. Because these two dimensions are based on different numbers of items and score ranges, the raw mean differences should not be interpreted as directly equivalent magnitudes of change. In addition, descriptive statistics indicated that posttest critical thinking scores in the experimental group approached the upper limit of the scale (*M* = 2.75, SD = 0.52, maximum possible score = 3.00), suggesting a possible ceiling effect. This restricted range may have limited sensitivity to further change on the critical thinking dimension and should be taken into account when interpreting both group differences and follow-up patterns. No comparable ceiling pattern was observed for the mathematical thinking dimension.

The *third research question* examined what pattern of short-term change, if any, was observed in life skills and computational thinking scores within the experimental group over the three-week follow-up period.

Follow-up analyses were based on the subset of experimental-group children with complete posttest and follow-up data (*n* = 110), as two children in the experimental group were absent on the day of the follow-up assessment. To examine changes in life skills over time, a paired-samples *t*-test was conducted for the experimental group. The results showed no statistically significant difference between the posttest and the follow-up measurement (*t*(109) = −0.40, *p* = 0.688, Cohen’s d = −0.04), indicating a trivially small effect. This null result indicates that scores did not decline significantly within the three-week period following the intervention. It does not, however, constitute evidence that the posttest pattern was retained or maintained over time, as no control-group follow-up was available for comparison and the follow-up interval was brief.

**Figure 3:**
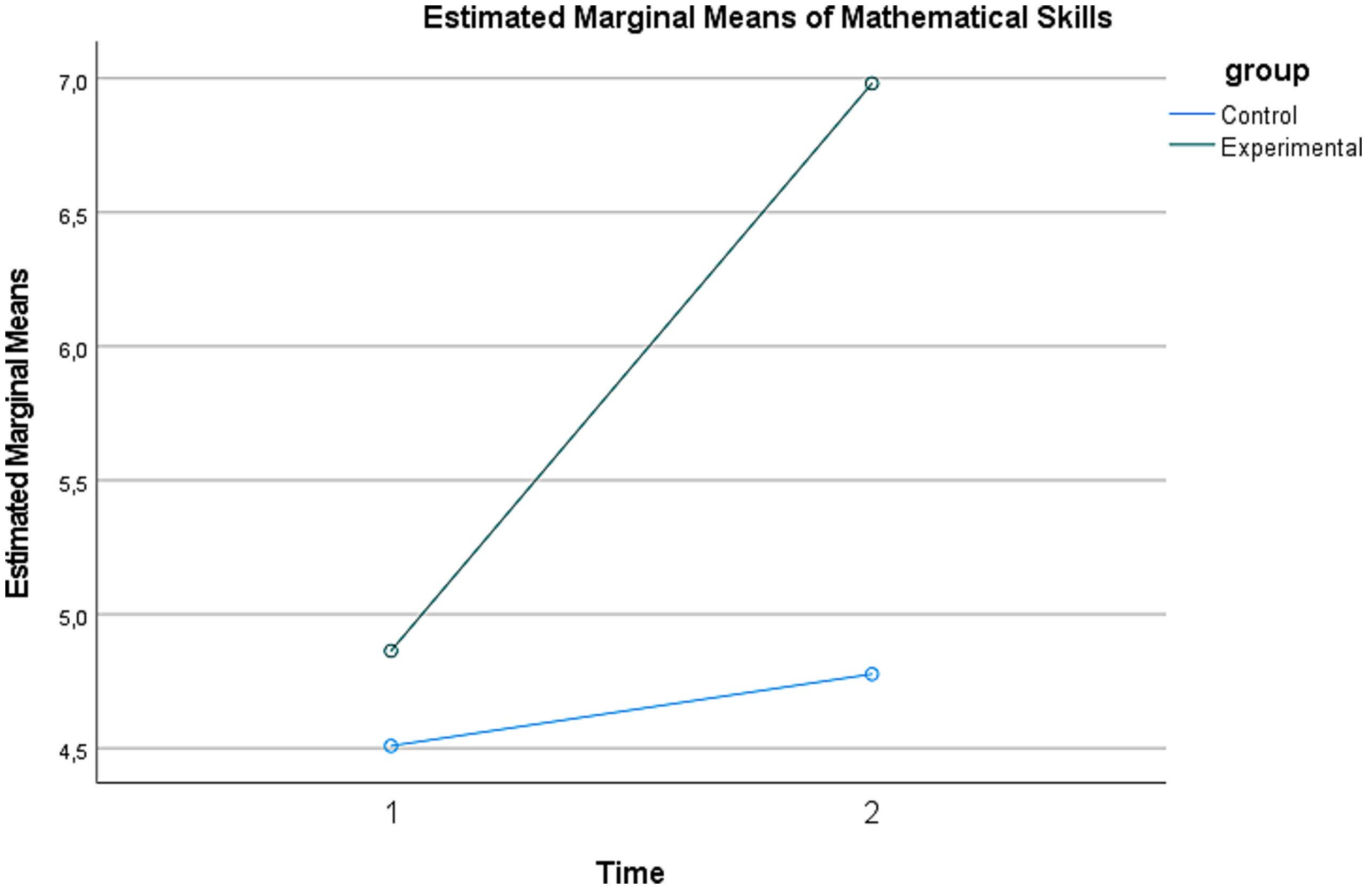
Mean change in mathematical thinking across groups.

To examine short-term maintenance in TechCheck-K outcomes within the experimental group, a repeated-measures multivariate analysis was conducted across time (posttest vs. follow-up) and skill dimension (mathematical thinking vs. critical thinking). The results showed that the main effect of time was not statistically significant (*V* = 0.016, *F*(1, 109) = 1.80, *p* = 0.182, ηp^2^ = 0.016). This null result indicates that TechCheck-K scores did not change significantly within the three-week post-intervention window in the experimental group. In addition, the non-significant interaction (*V* = 0.006, *F*(1, 109) = 0.61, *p* = 0.435, ηp^2^ = 0.006) indicates that this short-term pattern was similar across the two dimensions. These findings should be interpreted narrowly: the absence of a significant decline within the experimental group over 3 weeks does not constitute evidence of sustained program effects, particularly given the absence of control-group follow-up data and the restricted score range of the critical thinking subscale (Figure 3).

## 4 Discussion

Contemporary research indicates that sustainability-focused and nature-based pedagogies in early childhood contribute not only to cognitive domains such as executive functions and attention but also to socio-emotional development, including self-regulation, collaboration, and reductions in problem behaviors (Ardoin and Bowers, 2020; Kuo et al., 2019; Prins et al., 2022; Ulset et al., 2017). In line with this literature, the present study examined whether participation in the Sustainable Environmental Education Program (SEEP) was associated with differences in young children’s life skills and computational thinking-related scores, as well as what pattern of short-term posttest-to-follow-up change was observed within the experimental group. The findings were discussed in relation to relevant theoretical and empirical studies.

### 4.1 Life skills and Research Question 1

The findings related to the first research question indicate that, relative to the control group, children in the experimental group showed a statistically significant increase in teacher-reported life skills scores from pretest to posttest. This pattern is consistent with the possibility that sustainability-oriented learning experiences may be associated with broader life skill development; however, causal claims are not warranted given the quasi-experimental design, the absence of an attention-matched control condition, and the reliance on teacher-reported rather than independently verified outcomes. Recent empirical studies are also consistent with these findings, showing that participation in nature-based early childhood programs is associated with improvements in self-regulation, executive functions, attention, and classroom learning behaviors, and that these effects may be particularly pronounced among disadvantaged children (Ernst and Stelley, 2024; Ulset et al., 2017). Nevertheless, systematic reviews caution that while the majority of studies report positive relationships between nature-based environmental education and self-regulation, socio-emotional skills, and cognition, methodological heterogeneity and measurement diversity limit the certainty of the evidence (Ardoin and Bowers, 2020; Johnstone et al., 2022). In line with this, the present findings are consistent with prior studies suggesting that direct interaction with nature within a structured educational context may be associated with life skill development. However, the mechanisms underlying this pattern—whether through processes related to self-regulation, collaboration, or other factors—cannot be inferred from the current design and data. In sum, the life skills findings are best interpreted as associative and preliminary. The observed group pattern is consistent with the intervention hypothesis, but multiple alternative explanations cannot be ruled out. These findings should be interpreted in light of several methodological considerations. First, because the intervention was delivered at the classroom level but analyzed at the individual level without multilevel modeling, standard errors may be underestimated and statistical significance may be overstated. The use of robust multivariate test statistics and adjusted significance thresholds in some analyses provides only limited protection and does not fully compensate for unmodeled clustering. Second, life skills were assessed via teacher report, introducing potential for bias. Teachers were not blind to group assignment and may have been influenced by knowledge of the intervention or differential expectations. Third, the control group received standard preschool education but not an attention-matched control activity, leaving open the possibility that observed effects stem from novelty, researcher attention, or increased hands-on activity rather than sustainability content per se. Fourth, the time-of-day scheduling difference (experimental in morning, control in afternoon) may have introduced a confound, as children’s attention and energy can vary across the day. These limitations necessitate cautious interpretation.

### 4.2 Computational thinking and Research Question 2

The second research question focused on computational thinking-related outcomes, particularly mathematical and critical thinking. The findings indicate that, relative to the control group, children in the SEEP condition showed more favorable pretest-to-posttest patterns on these TechCheck-K dimensions; however, these findings should be interpreted as associative rather than conclusively causal in light of the quasi-experimental design and the classroom-level allocation of participants. This result is consistent with studies suggesting that nature-based and sustainability-oriented early childhood programs can support children’s mathematical school readiness. For example, a Finnish study reported that children attending nature preschools demonstrated parent-reported language and mathematics school readiness skills comparable to those of peers in typical preschools (Savolainen, 2025). Similarly, an ethnographic evaluation of a Forest School program in England found that three-year-old children made progress in the ‘mathematics’ domain of the Early Years Framework (de Cronin Chavez et al., 2024).

The development of mathematical skills is not solely dependent on content knowledge but also on cognitive processes that support computational thinking, notably executive functions (EF) such as working memory, inhibitory control, and cognitive flexibility. Although executive functions were not directly measured in the present study, prior research indicates that nature-based preschool experiences are associated with improvements in EF (Ernst and Stelley, 2024). It is conceivable that the hands-on, problem-solving structure of SEEP activities engaged executive function processes in ways that may have been associated with the observed score differences on the TechCheck-K. However, this interpretation is entirely speculative in the absence of direct EF measurement and cannot be supported by the current data. The observed improvements in mathematical thinking may reflect multiple mechanisms, including increased familiarity with quantitative concepts through nature-based activities, enhanced pattern recognition through repeated observation of natural phenomena, or simply increased engagement and motivation. Future research should include EF measures to test whether these processes mediate the relationship between sustainability education and computational thinking, and should explore alternative explanatory pathways.

The observed pattern in the TechCheck-K critical thinking dimension may be consistent with the view that sustainability-oriented activities can support reasoning and discussion in meaningful contexts. For instance, the Swedish “Systematic Child Talks” approach enabled preschoolers to engage in evidence-based discussions about sustainability, fostering justification, argumentation, and perspective-taking skills (Engdahl et al., 2023). Such dialogic pedagogies provide opportunities for children to weigh and compare multiple viewpoints, while nature-based play environments promote pattern recognition, spatial reasoning, and problem solving—all essential for CT (Speldewinde, 2022). Learning through direct contact with the natural world has been theorized to support understanding of abstract concepts and to encourage creative problem solving in the face of environmental challenges, although the evidence for these specific pathways in early childhood remains limited. Although the current findings are consistent with these theoretical and empirical strands, the broader literature base remains relatively small, and further, more comprehensive research is required to confirm and deepen the evidence linking sustainability education and CT in early childhood. Interpretation of the critical thinking findings also requires caution because this subscale includes only three items and experimental-group posttest scores approached the upper limit of the scale, suggesting a possible ceiling effect. As a result, the critical thinking dimension may have had limited sensitivity to further differentiation or posttest-to-follow-up change.

### 4.3 Short-term follow-up within the experimental group and Research Question 3

The third research question examined what pattern of short-term change, if any, was observed in life skills and TechCheck-K scores within the experimental group over the three-week follow-up period. As reported in the Results section, no statistically significant change in life skills or TechCheck-K scores was observed within the experimental group across this brief interval. These null results should not be interpreted as evidence of long-term retention or as confirmation that program-associated score differences were maintained over time. The follow-up period was short, the analyses were conducted within a single group without control-group follow-up data, and the critical thinking subscale showed a restricted score range with possible ceiling effects. Taken together, these constraints mean that the follow-up data provide only limited information about whether the observed posttest patterns would persist over more meaningful time periods. Future studies should include longer follow-up intervals and collect follow-up data in both experimental and control groups in order to evaluate durability more appropriately.

### 4.4 Contribution to the literature

This study adds to the still limited body of research examining life skills and computational thinking-related outcomes together within sustainability-oriented early childhood education. In this respect, it suggests that sustainability-based activities may be associated with selected socio-emotional and cognitive outcomes beyond environmental themes alone. The study may therefore offer a useful perspective for future research seeking to examine these domains in an integrated way.

The present findings are consistent with the possibility that sustainability-oriented pedagogies may be associated with selected life skill and cognitive outcomes in preschool settings. However, these results are exploratory and should not be taken as a basis for direct practice or policy recommendations in the absence of confirmatory evidence from more rigorous designs. If replicated through stronger designs, such findings would be consistent with UNESCO’s (2017) view of early childhood education as a context for fostering broader competencies. Future research with stronger designs and teacher-delivered implementations is needed before drawing practice or policy implications with confidence (Siraj-Blatchford and Pramling Samuelsson, 2015).

### 4.5 Limitations and future directions

This study has several limitations that should be considered when interpreting the findings. It is important to interpret these findings within the constraints of school-based early childhood intervention research. In preschool settings, random assignment of individual children to conditions is often difficult to implement without disrupting naturally formed classroom groups or increasing the risk of treatment diffusion across children. For this reason, quasi-experimental designs with intact comparison groups remain common in this area of research. Nevertheless, such designs permit only limited causal inference, and the present findings should therefore be interpreted as preliminary and in need of confirmation through studies with stronger cluster-level designs. First, the intervention was assigned at the classroom level, but the analyses were conducted at the individual level because the limited number of classrooms did not permit multilevel modeling. As a result, classroom-level dependency may not have been fully accounted for, and the findings should therefore be interpreted with caution. Second, life skills were assessed through teacher ratings. Although teachers were able to observe children across everyday classroom contexts, reliance on a single informant may have introduced subjective bias. In addition, teachers were aware of classroom participation in the intervention, and expectancy effects therefore cannot be ruled out. Third, the control group continued with the regular preschool curriculum but did not receive an alternative structured intervention matched for researcher attention, hands-on activity, or novelty. Accordingly, the observed group differences cannot be attributed solely to the sustainability content of the program with complete certainty. Fourth, the experimental and control classrooms were scheduled at different times of day in order to reduce interaction between conditions. Although this arrangement helped limit contamination, it may also have introduced a time-of-day confound. Fifth, the study did not include direct measures of teacher characteristics, classroom climate, or instructional quality. Because the study used a classroom-allocated quasi-experimental design rather than individual random assignment, unmeasured teacher- and classroom-level differences may have contributed to the observed outcomes. In addition, the critical thinking dimension of TechCheck-K includes only three items, resulting in a restricted score range that may have increased susceptibility to ceiling effects and limited sensitivity to change, particularly at posttest and follow-up. Sixth, the sample was drawn from a single mid-sized city in Türkiye, which may limit the generalizability of the findings to other geographic, cultural, and educational contexts. Finally, the intervention was delivered by trained members of the research team rather than by regular classroom teachers. Although this supported implementation consistency, it may limit conclusions about everyday classroom feasibility and scalability.

These limitations also point to several directions for future research. Subsequent studies should include larger numbers of classrooms and analytic approaches that more fully account for nested data structures. Future research would also benefit from the use of multiple methods and multiple informants, including direct observation, parent report, and performance-based assessments. In addition, active control conditions matched for structure and intensity would help clarify whether observed effects are related specifically to sustainability content or to broader features of the intervention. Longer follow-up periods in both experimental and control groups are needed to evaluate the durability of the findings over time. It would also be valuable to examine teacher-delivered implementations under routine preschool conditions, to assess feasibility and scalability, and to replicate the study in diverse cultural and educational settings. Finally, future studies may expand the range of outcomes by including variables such as environmental understanding, classroom participation, and other developmentally relevant indicators.

## 5 Conclusion

This classroom-based quasi-experimental study provides preliminary, exploratory evidence that participation in a structured sustainability-based environmental education program may be associated with more favorable posttest patterns in teacher-reported life skills and performance-based computational thinking scores among preschool-aged children. The Sustainable Environmental Education Program (SEEP), delivered over 10 weeks through hands-on activities with natural and recyclable materials, was associated with more favorable posttest outcomes than standard preschool programming. Within the experimental group, no statistically significant change was observed over the three-week posttest-to-follow-up interval; however, this brief single-group follow-up should not be interpreted as evidence of durable program effects. However, multiple methodological limitations—most notably classroom-level assignment analyzed with individual-level models due to insufficient cluster sample size, reliance on single-informant measurement for life skills, absence of an attention-matched control condition, time-of-day scheduling differences, restricted score range in the critical thinking subscale, and short-term follow-up only in the experimental group—necessitate cautious interpretation. These findings should be considered hypothesis-generating rather than conclusive. They suggest that sustainability education may offer a promising context for supporting broader cognitive and socio-emotional outcomes alongside sustainability-related learning experiences, but this possibility requires more rigorous testing before warranting confident endorsement or large-scale implementation.

The detailed description of SEEP’s theoretical foundations, pedagogical approach, and specific activities provides a resource for other researchers and practitioners seeking to develop or evaluate similar programs. However, adaptation to local contexts, cultural norms, available resources, and teacher capacity will be essential.

In the context of urgent environmental challenges and the need to prepare children for an uncertain future, early childhood sustainability education represents a potentially valuable pedagogical approach. However, transforming potential into demonstrated effectiveness requires methodological rigor, conceptual clarity, and sustained empirical inquiry. This study contributes a documented and methodologically transparent step in that direction. At the same time, the quasi-experimental design, classroom-level clustering not modeled in the primary analyses, reliance on teacher-reported life skills ratings, and short follow-up limited to the experimental group indicate that the findings should be interpreted cautiously and require confirmation through more rigorous future research.

## Data Availability

The raw data supporting the conclusions of this article will be made available by the authors, without undue reservation.
